# Characterization of VitE-TPGS Micelles Linked to Poorly Soluble Pharmaceutical Compounds Exploiting Pair Distribution Function’s Moments

**DOI:** 10.3390/pharmaceutics17040431

**Published:** 2025-03-27

**Authors:** Liberato De Caro, Thibaud Stoll, Arnaud Grandeury, Fabia Gozzo, Cinzia Giannini

**Affiliations:** 1 Istituto di Cristallografia, Consiglio Nazionale delle Ricerche, Via Amendola 122/O, 70125 Bari, Italy; cinzia.giannini@cnr.it; 2 Excelsus Structural Solutions (Swiss) AG, Park Innovaare, Parkstrasse 1, 5234 Villigen, Switzerland; thibaud.stoll@excelsus2s.com (T.S.); fabia.gozzo@excelsus2s.com (F.G.); 3 Novartis Pharma AG, Technical Research and Development, Material Science, Novartis Campus, Virchow 6.3.231, 4056 Basel, Switzerland; arnaud.grandeury@novartis.com

**Keywords:** drug delivery systems, surfactant, Small-Angle X-ray Scattering, micelles

## Abstract

**Background:** Micelles have attracted significant interest in nanomedicine as drug delivery systems. This study investigates the morphology of micelles formed by the D-α-tocopherol polyethylene glycol 1000 succinate (VitE-TPGS) surfactant in the presence and absence of, respectively, a poorly soluble pharmaceutical compound (PSC), i.e., Eltrombopag (0.08 wt%) and CaCl_2_ (0.03 wt%). The aim was to assess the micelles’ ability to solubilize the PSC and potentially shield it from Ca^2+^ ions, simulating in vivo conditions. **Methods:** For this purpose, we have developed a novel theoretical approach for analyzing Pair Distribution Function (PDF) data derived from Small-Angle X-ray Scattering (SAXS) measurements, based on the use of PDF’s moments. **Results:** Our spheroid-based model was able to characterize successfully the micellar morphology and their interactions with PSC and CaCl_2_, providing detailed insights into their size, shape, and electron density contrasts. The presence of PSC significantly affected the shape and integral of the PDF curves, indicating incorporation into the micelles. This also resulted in a decrease in the micelle size, regardless of the presence of CaCl_2_. When this salt was added, it reduced the amount of PSC within the micelles. This is likely due to a decrease in the overall PSC availability in solution, induced by Ca^2+^ ions. **Conclusions:** This advanced yet straightforward analytical model represents a powerful tool for characterizing and optimizing micelle-based drug delivery systems.

## 1. Introduction

Micelles have attracted significant attention in nanomedicine, particularly for their potential as drug delivery systems [1,2,3,4]. These nanoscale structures, typically formed by the self-assembly of amphiphilic molecules in an aqueous environment, usually surfactants, exhibit a core–shell architecture that is highly conducive to encapsulating hydrophobic drugs. The core of the micelle provides a hydrophobic environment suitable for the entrapment of water-insoluble drugs, while the hydrophilic shell stabilizes its structure in biological fluids, thereby enhancing the solubility, bioavailability, and targeted delivery of therapeutic agents. Among the various factors that influence the efficacy of micelles as drug carriers, their morphology—encompassing both size and shape—plays a pivotal role [5].

The morphology of micelles directly impacts their drug-loading capacity and stability, in particular upon dilution, circulation time, and biodistribution, which are crucial parameters for effective drug delivery [6,7,8,9,10,11]. The size of the micelles is particularly important because it determines the ability of the micelles to pass through biological barriers, including cellular membranes, and to evade the reticuloendothelial system (RES), which is responsible for clearing foreign particles from the bloodstream. Micelles ranging in size from approximately 10 to 100 nm are often ideal for prolonged circulation, as well as for enhancing permeability and retention (EPR) effect, a phenomenon that enables nanoparticles to accumulate preferentially in tumor tissues due to their leaky vasculature and inefficient lymphatic drainage [7].

Although micelles have been traditionally approximated as spherical systems, recent research has revealed the existence of other shapes, such as ellipsoidal, rod-like, worm-like or even disk-like structures. These different shapes are mainly due to the structure of the micelle-forming surfactants used and to the characteristics of the surrounding environment (temperature, pH, and chemical composition). Non-spherical micelles have been shown to exhibit different cellular uptake mechanisms, circulation times, and biodistribution patterns compared to their spherical counterparts. For instance, they may exhibit prolonged circulation times due to reduced recognition and clearance by the RES, or they may demonstrate enhanced tissue penetration and cellular uptake via mechanisms that are less dependent on size, influencing the drug release profile, which is a critical factor affecting therapeutic efficacy. The internal structure of micelles, which is influenced by its size and shape, dictates the distribution of the drug within the micellar core and the interactions between the drug and the micelle-forming molecules. This, in turn, affects the rate at which the drug is released from the micelles. For example, smaller micelles may release their encapsulated drug more rapidly due to a higher surface-area-to-volume ratio, while larger or elongated micelles may provide a more sustained release profile. The ability to fine-tune the release kinetics through careful control of micelle morphology is particularly important for applications requiring a controlled and sustained release of the drug over time, such as in chronic disease management and chemotherapy. In these cases, minimizing side effects while maximizing therapeutic efficacy is paramount. Additionally, sustained-release drug delivery systems can improve the patients’ quality of life by reducing dosing frequency, easing the burden of multiple daily administrations, and enhancing treatment adherence. This benefit is particularly important for patients with chronic conditions, as it ensures consistent medication delivery without the stress of managing multiple daily doses.

The size and shape of micelles influence their interaction with cell membranes, their uptake by cells, and their intracellular trafficking pathways [12]. These factors are crucial for ensuring that the drug reaches its target within the body, whether that target is a specific tissue, organ, or subcellular compartment. For example, smaller micelles are more readily taken up by cells via endocytosis, while larger or non-spherical micelles may interact differently with cell membranes, potentially leading to alternative uptake pathways or enhanced retention within specific tissues.

For the above reasons, an accurate and precise measurement of the micelle size and shape is essential. Various analytical techniques, such as dynamic light scattering (DLS), transmission electron microscopy (TEM), and cryogenic electron microscopy (cryo-EM), are commonly used for this purpose. They each have distinct advantages and provide complementary information about the size, shape, and internal structure of micelles. For instance, DLS is widely used for determining the hydrodynamic diameter of micelles in solution, while TEM and cryo-EM enable direct visualization of micelle morphology, providing detailed insights about their shape and internal organization.

In addition to these conventional techniques, emerging methods such as Small-/Wide-Angle X-ray Scattering (SAXS/WAXS) and atomic force microscopy (AFM) are being increasingly utilized to obtain more comprehensive and high-resolution data on micelle morphology [12]. These advanced techniques are particularly valuable for gaining a deeper understanding of the structural features that influence drug loading and release. SAXS is particularly suitable for the characterization of micelles in solution, under real conditions, requiring limited sample preparation.

In 2023, we developed a novel theoretical approach for analyzing Pair Distribution Function (PDF) data derived from SAXS measurements, based on a spherical core–shell model for micelles [13]. This method enables the determination of the core–shell and the shell–buffer electron density contrasts, as well as the core and shell sizes of micelles, by modeling the PDF data. The approach has been validated by applying it to VitE-TPGS micelles [13]. As a further development, we have extended our approach to model spheroidal core–shell micelles [14]. This new approach represents a significant improvement, as it uses the first derivative of the PDF and analytical equations to resolve the structure of spheroidal core–shell micelles. By applying these equations, we can determine key structural parameters, including the micelle’s aggregation number, ellipticity, and electron density contrast between core and shell regions. The method was applied successfully to micelles formed by different surfactants, such as Polysorbate 20, Dodecyl phosphocholine, Sodium Dodecyl Sulfate, and VitE-TPGS.

In this work, assuming spheroidal-shaped micelles, we exploit nth-order moments of the PDF, with n ranging from 1 to 6, to perform the structural characterization of VitE-TPGS micelles both in the presence and absence of a poorly soluble pharmaceutical compound (PSC). In our study, the PSC was the active pharmaceutical ingredient Eltrombopag, a thrombopoietin receptor agonist, which is used to treat low blood platelet counts in adults with chronic immune (idiopathic) thrombocytopenia. It was approved by the FDA in 2008 [15]. Additionally, samples with and without CaCl_2_ in the buffer solution have been measured, to assess the potential role of micelles to prevent the interaction reported when the formulated drug was administered with high-calcium-content food [16]. Unlike the previous method [14], which solved the micelle structure with a hybrid approach, i.e., partially graphical (first derivative of the PDF) and partially analytical, we present here a fully analytical method. It is described in Section 2 and Appendix A, while the experimental data are summarized in Section 3 and discussed in Section 4.

## 2. Analytical Method

For a two-component spheroidal micelle, we can derive the analytical expression of the following integrals, for any *n* = 1, …, N; they are reported explicitly in Appendix A:

(1) $$R^{n}=\left(\int r^{n}\left(\rho \left(r\right)-\rho_{s}\right)d^{3}r\right)/\left(\int \left(\rho \left(r\right)-\rho_{s}\right)d^{3}r\right).$$

Here, $\rho_{s}$ = solvent electron density, $\rho \left(r\right)=\rho_{E}$, the electron density inside the core, and $\rho \left(r\right)=\rho_{P}$, the electron density inside the shell. These integrals are a measure of the atomic electron density within the micelle as a function of the nth power distance *r*^n^, like raw moments for a probability distribution calculated from zero and not with respect to the mean (central moments).

We can compare the analytical expression obtained by Equation (1) with the corresponding nth r-power integral of the PDF, derived from the experimental SAXS data:

(2) $$R_{\mathrm{PDF}}^{n}\;=\frac{\int_{0}^{D_{max}}PDF\left(r\right)r^{n}dr}{2^{n-1}\int_{0}^{D_{max}}PDF\left(r\right)dr}.$$

Here $D_{max}$ is the maximum distance in the PDF. In this way, allowing *n* to range from 1 to N, we can derive a set of N independent equations with the following unknowns: the ellipticity ε; the core-solvent electron contrast $\Delta \rho_{ES}=\rho_{E}-\rho_{s}$; the shell-solvent electron contrast $\Delta \rho_{PS}=\rho_{P}-\rho_{s}$; the micelle size $D_{max}$; and the shell radius $R_{\mathrm{SH}}$. Since we need at least 5 independent equations to determine the 5 unknown parameters defining the micelles’ structure, we can also overdetermine the solution by solving a set of N equations with N ≥ 5, by means of a least-square-minimum approach, as described in Appendix A. If N > 5, we would have an overdetermined system of equations.

## 3. Results

The micelles studied in this work are formed by VitE-TPGS surfactant monomers, with and without the presence in solution of a PSC, in order to study the level of incorporation of this compound into the micelles. SAXS data were collected at the SAXS Lab Sapienza with a Xeuss 2.0 Q-Xoom system (Xenocs SA, Grenoble, France). See details in [13]. We have considered four cases, summarized in Table 1. The nominal pH is 6.8. Samples 1 and 2 have been prepared with PSC, with and without CaCl_2_ added to the buffer solution. Samples 3 and 4 have been prepared with PSC, with and without CaCl_2_ added to the buffer. The nominal concentrations of VitE-TPGS and of the potassium phosphate buffer were kept constant across all experiments at 0.415% *w*/*w* and 50 mM, respectively. In subsequent solutions, concentration in PSC was maintained at 0.08% (*w*/*w*) and calcium chloride at about 0.03% (*w*/*w*). It corresponds to quite a large excess of calcium cations vs. PSC. The aim was to simulate digestive tract conditions, where high concentrations of Ca^2+^ from calcium-rich foods may be present and negatively affect the bioavailability and solubility of active pharmaceutical ingredients, and to evaluate the potential protective role of micelles. Samples 1–4 have been prepared according to the following protocol to maximize the sameness of the solution, minimizing experimental variability.

A buffer solution was prepared by mixing 682.7 mg of potassium hydrogen phosphate (K_2_HPO_4_) and 2.26 mmol of NaOH (added as 22.6 g of a 0.4 wt% solution) in Milli-Q water, to reach a final volume of 100 mL. The solution was stirred until a stable pH value was reached. The targeted value was 6.80 while the actual measured value was 6.81. The buffer solution was used to prepare all 4 samples, to ensure the same pH value. To prepare sample 1, 82.6 mg of VitE-TPGS was dissolved in 19.9 g of the pH 6.8 (50 mM) buffer solution to reach a concentration of 0.415 wt%. The sample was stirred at 500 rpm for 43 min to achieve complete dissolution. No significant variation was observed for the pH, in comparison to the buffer solution (as expected, since VitE-TPGS has no acidity/basicity). The final pH value was 6.87, before filtration. An aliquot of this sample was then filtered four times through Axiva sterile cellulose acetate syringe filters with pore diameters of 200 nm to remove any undissolved particulate matter and ensure a homogeneous solution. SAXS data collected on the unfiltered and filtered samples were superimposable, indicating that no undissolved surfactant in the form of particulate greater than 200 nm was present in the unfiltered sample. To prepare sample 2, 0.55 mg of CaCl_2_ was dissolved in 2.4 g of the sample 1 solution, corresponding to 0.024 wt% of CaCl_2_, and the solution was stirred at 500 rpm for 20 min. No significant variation was observed for the pH, as expected, since CaCl_2_ has no particular acidity or basicity. To prepare sample 3, 8.0 mg of PSC was added to 10 g of the sample 1 solution to reach a concentration of 0.08 wt%. The solution was stirred at 500 rpm for 60 min to ensure complete dilution. After stirring, no significant deviation towards higher pH values was observed thanks to the buffer capacity. The final pH value was 6.91. To prepare sample 4, 0.8 mg of CaCl_2_ was added to 2.8 g of the sample 3 solution, corresponding to a concentration of 0.415 wt% of VitE-TPGS, 0.08 wt% of PSC, and 0.029 wt% of CaCl_2_. The solution was stirred at 500 rpm for 20 min. No significant variation was observed for the pH. The final pH value after filtration was 6.84. All preparations were performed at 25 °C. Table 1 summarizes the composition of the 4 samples.

**Figure 1:**
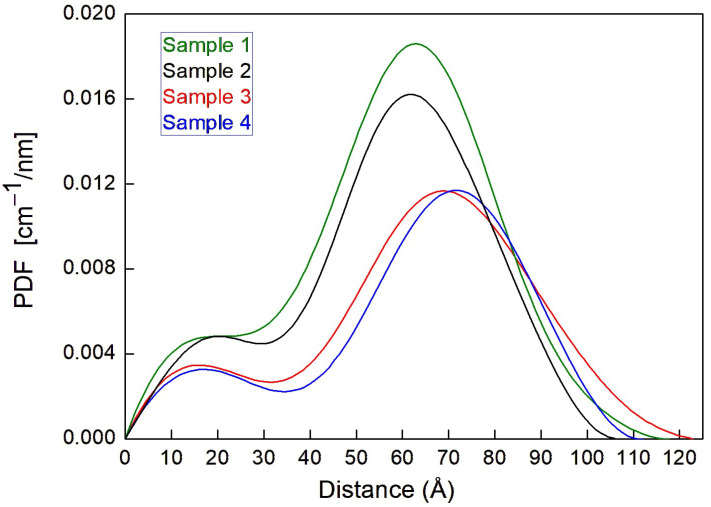
PDF curves, expressed on a quantitative scale, obtained for samples 1, 2, 3, and 4.

The PDF curves, obtained for these samples, are shown in Figure 1; they describe the SAXS macroscopic cross-section as a function of the atom–atom distances r.

The PDFs, calculated by the software GNOM 47.5 [17], have been rescaled so that the integral of the PDFs plotted in Figure 1 is equal to the corresponding *I*(0) [cm^−1^] value, i.e., the SAXS macroscopic cross-section for the whole sample extrapolated to the scattering vector value q = 0. Figure 1 shows that the presence of the PSC significantly affects the shape and the integral of the PDF curve, which indicates that some PSC molecules have been incorporated into the micelles. The presence of CaCl_2_ in solution reduces the integral of the macroscopic cross-section (cf green curve versus black curve in Figure 1), indicating that a smaller quantity of PSC could be incorporated into the VitE-TPGS micelles. A possible explanation for the observed reduction of the amount of PSC in the micelles (and possibly also outside, in the same buffer solution) in the presence of CaCl_2_ is the occurrence of some specific interactions, eventually exemplified by the drug. Cation interactions are indeed known to reduce the bioavailability of the drug [18].

After having determined $\varepsilon ,\;\Delta \rho_{ES},$ $\Delta \rho_{PS},$ $D_{M}$, $R_{SH}$, from Equations (1) and (2), via a least-square-minimum approach discussed in Appendix A, we can evaluate the aggregation number of monomers inside the micelles ($N_{agg}$), the number of water molecules inside the shell ($N_{H2O}),\;$and the number of PSC molecules per monomer inside the micelles ($x_{PSC}=N_{PSC}$/$N_{agg}$). All formulas are provided in Appendix A. Table 2 summarizes the micelles’ parameters obtained for the 4 samples.

**Figure 2:**
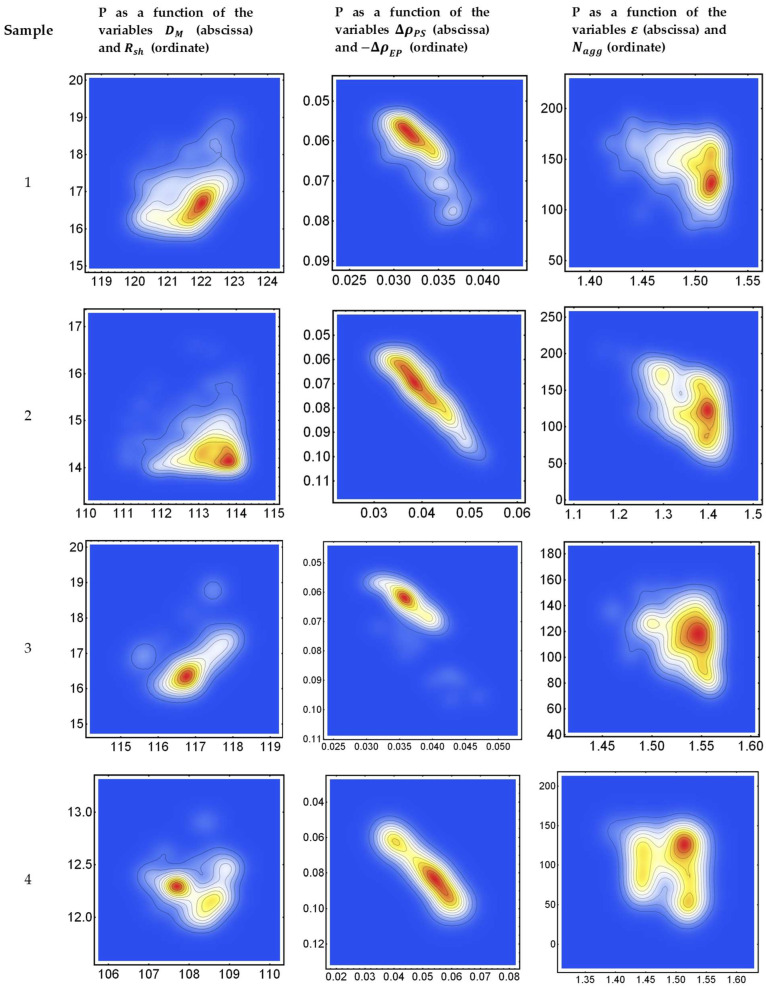
(**Left**): Probability density function (P), associated with the histograms of LRS values obtained from Equation (A15), for samples 1, 2, 3, and 4, as a function of the variables $D_{M}$ and $R_{sh}$, expressed in Å. (**Center**): Probability density function, associated with the histograms of LSR values obtained from Equation (A15) as a function of the variables $\Delta \rho_{Ps}$ and $-\Delta \rho_{EP}=-\Delta \rho_{E}+\Delta \rho_{P}$, expressed in *n_e_*/Å^3^. (**Right**): Probability density function, associated with the histograms of LSR values obtained from Equation (A15) as a function of the variables ε and $N_{agg}$, expressed in dimensionless units. Ten contours have been determined, represented as 10%, 20%, …, 80%, 90% levels of the histogram maximum. The red color is associated with 90% of the maximum.

Figure 2 shows, for the four samples, the probability density function calculated by the SmoothDensityHistogram function implemented in Mathematica (Wolfram), associated with the histograms of least-square-root values (LSR) (cf Equation (A15)) obtained by the least-square approach discussed in Appendix A. We have set ten contour levels in the probability density function (P) at 10%, 20%, …, 80%, 90% of the histogram maximum. The red color is associated with 90% of the maximum. The left column on Figure 2 shows P as a function of the variables $D_{M}$ and $R_{sh}.\;$The enter column shows P as a function of the variables $\Delta \rho_{PS}$ and $-\Delta \rho_{EP}=-\Delta \rho_{ES}+\Delta \rho_{PS}.\;$The right column shows P as a function of the variables ε and $N_{agg}$.

It is interesting to note that in Figure 2, secondary maxima are occasionally observed in the two-dimensional P histograms. This is particularly evident for sample 4, suggesting that the presence of both PSC and CaCl_2_ could cause a certain level of variability in the micelles’ structure, which, in turn, could imply the occurrence of a certain level of interaction between the PSC and Ca^2+^ cations in solution during the formation of micelles. Further investigations would be needed to validate this hypothesis.

## 4. Discussion

The comparison of the $D_{max}$ and $R_{g}$ values from Table 2 shows that the addition of PSC leads to the formation of smaller micelles, whether CaCl_2_ is present or not. A similar effect can be seen on the shell size (R_SH_) and the equatorial core radius. These observations are consistent with those made in our previous work [13], while our new spheroidal model provides here a more detailed characterization of the micelle morphological changes.

**Figure 3:**
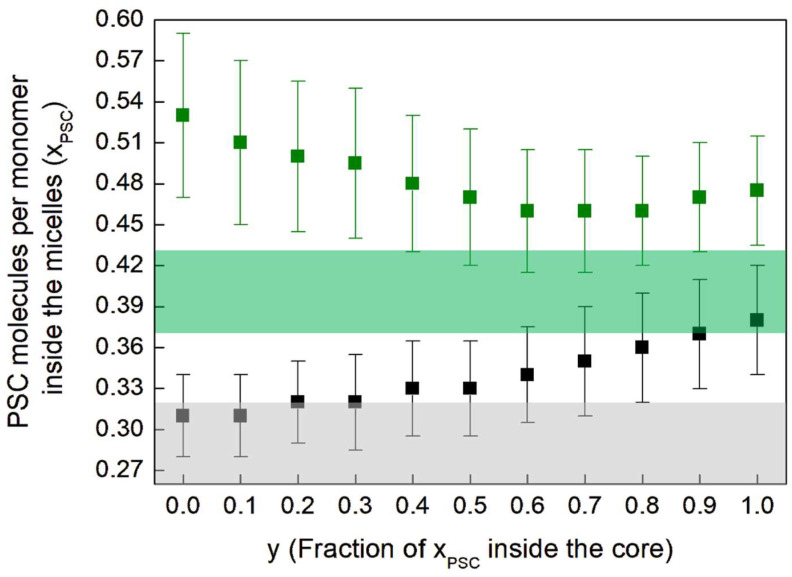
Number of PSC molecules $x_{PSC}\;$per monomer inside the micelles of sample 3 (green) and 4 (black) as a function of the fraction of $x_{PSC}$ inside the core (denoted as *y* in Equation (A18)).

In our previous work [13], we assumed spherical-shaped micelles, following the suggestions in [19]. Under this hypothesis, we estimated, for sample 3, $x_{PSC,3}=0.30\;\pm \;0.01$. By introducing, with *y*, the fraction of the PSC molecules per monomer inside the core (see Appendix A) and by relaxing the spherical-shape constraint by solving Equation (A22), we obtain the results shown in Figure 3, where $x_{PSC}$ is represented as a function of y for samples 3 and 4. For sample 3 we obtain $x_{PSC,3}$ ranging between 0.47 and 0.53, as a function *y*. This difference with respect to the result obtained in [13] is mainly due to both the different number of monomers aggregated into the micelles and the different micelle shape given by the 2 models (spherical vs. spheroidal). Indeed in [13], from the difference of the PDF curves obtained with and without PSC in solution, we had estimated, under the hypothesis of spherical-shaped micelles, the localization of these molecules inside the micelles, close to the linker region, and a concentration $x_{PSC,3}=0.30\;\pm \;0.01$. For spheroidal-shaped micelles, this approach is not possible, because differences in intensity of the PDF at the same distance from the center could also be due to both different number of aggregated monomers and to different ellipticity values, which, in turn, influence the distribution of distances in the PDF profile and the estimated value for $x_{PSC}$.

The colored bands in Figure 3 are the constraints for samples 3 (in green) and 4 (in grey), derived from Equation (A16) and developed in Equations (3) and (4) below. Indeed, from Equation (A16), it follows that the square difference of electrons per monomer, between the samples with and without PSC, must be equal to the difference of $I\left(0\right)/K$:

(3) $${\left[827+232x_{PSC,3}-10\frac{2327+554x_{PSC,3}}{29.9}\right]}^{2}N_{agg,3}-{\left[827-10\frac{2327}{29.9}\right]}^{2}N_{agg,1}\;={\left[I\left(0\right)/K\right]}_{3}-{\left[I\left(0\right)/K\right]}_{1};$$

(4) $${\left[827+232x_{PSC,4}-10\frac{2327+554x_{PSC,4}}{29.9}\right]}^{2}N_{agg,4}-{\left[827-10\frac{2327}{29.9}\right]}^{2}N_{agg,2}\;={\left[I\left(0\right)/K\right]}_{4}-{\left[I\left(0\right)/K\right]}_{2}.$$

The numerical values 827, 232, and 10 correspond to the number of electrons of a VitE-TPGS, PSC, and water molecule, respectively; 2326 , 554, and 29.9 are the molecular volumes of these 3 species in Å^3^ [13]. From Equation (3), we have, for samples 3 and 4, $x_{PSC,3}=0.40\;\pm \;0.03$ and $x_{PSC,4}=0.29\;\pm \;0.03$, respectively, which are represented as colored bands in Figure 3.

For sample 3, the middle value of the green band is 0.40; the upper value is 0.43 and the lower value is 0.37. We have obtained the grey band, for sample 4, similarly. The grey and green bands have to be compared with the black and green squares of Figure 3, respectively. The squares that are within the colored bands are possible solutions of all equations. By comparing these values with those obtained from Equation (A22), reported as squares in Figure 3, we obtain that for sample 3, the fraction of $x_{PSC}$ inside the core should be $y_{3}=0.65\;\pm \;0.15$. For sample 4, we have $y_{4}=0.35\;\pm \;0.35$, a range that includes also 0. Therefore, in the presence of CaCl_2_ in buffer solution, the PSC molecules are much less segregated into the core and not isolated from the external environment with respect to the hydrophobic core. They are almost all in the shell, still in interaction with the buffer, due to its hydration. This result is also supported by the very low shell hydration of the micelles of sample 4 (see Table 2), because the available space in the shell is effectively already occupied by the PEG-1000 tails and the PSC molecules. Conversely, in the absence of CaCl_2_ in solution, the PSC molecules are almost all localized in the core and, partially, in the region of the linker, being hence much better isolated from the buffer.

As calculated in Appendix A, the ratio of N_PSC_ and N_mon_ in sample 3 is about 2/3. The addition of PSC in the solution leads to micelles of a smaller diameter, as can be seen from the results of Table 2 for samples 3 and 4. The addition of CaCl_2_ in the solution reduces the hydration of the PEG-1000 shell too. Moreover, in the presence of PSC, the CaCl_2_ in solution leads to the formation of micelles with a smaller fraction of PSC linked to them, as can be clearly seen in Figure 3. This observation could be explained by the fact that the presence of Ca^2+^ in solution reduces the incorporation of PSC molecules into the hydrophobic core of the micelles. Additionally, from the $x_{PSC}\;$and the N_agg_ values (Table 2), we can estimate the total number of PSC molecules per micelle to 47 in sample 3 and 36 in sample 4. These combined results suggest that the presence of CaCl_2_ in the buffer solution reduces the incorporation of PSC molecules both specifically into the hydrophobic core and in total into the micelles. One possible mechanism could be the overall reduction of available PSC in solution, induced by the presence of Ca^2+^ ions.

## 5. Conclusions

Under the assumption of a spheroidal model for micelles, we have developed a new fully analytical method, which exploits nth-order moments of the PDF derived from SAXS measurements to determine the micelles’ structural parameters. This model has been successfully applied to characterize the morphology of micelles with and without the presence of a poorly soluble API (PSC) and CaCl_2_, respectively. We were able to confirm previous observations on the impact of PSC based on a simple spherical model, although now with many more details, thanks to our new spheroidal model. With this model, we were also able to estimate the relative fractions of the PSC incorporated in the core and in the shell of the micelles. The model also reveals that the presence of CaCl_2_ reduces PSC incorporation, both within the hydrophobic core and the micelle as a whole. This advanced yet straightforward analytical model offers a powerful tool for characterizing micellar systems and guiding the development of optimized drug delivery strategies.

Future experiments, where the micelles’ morphology is characterized with our model as a function of the CaCl_2_ concentration, could help us further understand the role of Ca^2+^ in influencing the micelles’ shape and size, and to which extent micelles can play a protective role for the PSC molecules. These studies could obviously be extended to other types of surfactants and PSC. The results of such in vitro studies could provide useful insights to understand in-vivo mechanisms. They could then help design drug delivery systems that can enhance the bioavailability of poorly soluble APIs and protect them against the detrimental effect of Ca^2+^ cations in vivo, which are known to impact significantly the absorption and effectiveness of some pharmaceuticals administered orally.

## Figures and Tables

**Table 1:** Composition of samples 1–4, containing the VitE-TPGS surfactant in the buffer solution, without and with a poorly soluble compound (PSC), as well as without and with CaCl_2_.

Sample		VitE-TPGS	PSC	CaCl_2_	pH Measured
1	19.9 g of buffer 50 mM	82.6 mg (0.415 wt%)	No	No	6.87
2	2.4 g of sample 1	Yes (0.415 wt%)	No	0.55 mg (0.024 wt%)	6.82
3	10 g of sample 1	Yes (0.415 wt%)	8 mg (0.08 wt%)	No	6.91
4	2.8 g of sample 3	Yes (0.415 wt%)	Yes (0.08 wt%)	0.8 mg (0.029 wt%)	6.84

**Table 2:** Shell size ($R_{SH}$), maximum micelle size ($D_{max}$), ellipticity (ε), $\Delta \rho_{ES}=\Delta \rho_{EP}+\Delta \rho_{PS}$, $\Delta \rho_{PS}$, $N_{agg},$ $N_{H2O}$/$N_{agg}$ obtained from the analysis of the PDF’s moments of VitE-TPGS micelles. $c_{mon}$ = 4.1 mg/mL. *cmc* = 0.02 mg/mL. $R_{g}\;$is the gyration radius. *I*(0) is the PDF integral on an absolute scale. For prolate micelles, the minimum core size is in the equatorial plane (equatorial core radius).

Sample	$R_{SH}$ (Å)	$D_{max}$ (Å)	Equatorial Core Radius $(D_{max}$ − $2R_{SH}$)/2ε (Å)	$\Delta \rho_{ES}$ (*n_e_*/Å^3^)	$\Delta \rho_{PS}$ (*n_e_*/Å^3^)	ε	$N_{agg}$	$N_{H2O}$ $/N_{agg}$	$R_{g}$(Å)	*I*(0) (cm^−1^)
1 (without PSC and CaCl_2_)	16.7 ± 0.1	122.0 ± 0.1	29.3 ± 0.2	−0.026 ± 0.001	0.032 ± 0.001	1.51 ± 0.01	127 ± 2	53 ± 4	47.3 ± 0.5	0.056 ± 0.001
2 (without PSC, with CaCl_2_)	14.2 ± 0.1	113.8 ± 0.1	30.5 ± 0.2	−0.031 ± 0.001	0.039 ± 0.001	1.40 ± 0.01	122 ± 3	36 ± 4	47.3 ± 0.4	0.055 ± 0.001
3 (with PSC, without CaCl_2_)	16.4 ± 0.1	116.7 ± 0.1	27.1 ± 0.2	−0.026 ± 0.001	0.036 ± 0.001	1.55 ± 0.01	118 ± 4	44 ± 4	43.5 ± 0.5	0.085 ± 0.001
4 (with PSC and CaCl_2_)	12.3 ± 0.1	107.7 ± 0.1	27.7 ± 0.2	−0.030 ± 0.001	0.055 ± 0.001	1.51 ± 0.01	125 ± 4	12 ± 5	42.8 ± 0.3	0.079 ± 0.001

## Data Availability

No new data were created or analyzed in this study. Data sharing is not applicable to this article.

## Abbreviations

The following abbreviations are used in this manuscript:

VitE-TPGS	D-α-tocopherol polyethylene glycol 1000 succinate
PSC	Poorly soluble pharmaceutical compound
PDF	Pair Distribution Function
SAXS	Small-Angle X-ray Scattering
WAXS	Wide-Angle X-ray Scattering
RES	Reticuloendothelial system
EPR	Enhanced permeability and retention
DLS	Dynamic light scattering
TEM	Transmission electron microscopy
Cryo-EM	Cryogenic electron microscopy
AFM	Atomic force microscopy
LSM	Least square minimum
PEG	Polyethylene glycol

## Appendix A

### Determination of the Micelles’ Structure by Evaluating the PDF’s Moments Through a Least-Square Approach

For two-component micelles with a prolate or oblate spheroid shape (ellipsoids of revolution around the polar axis), the electron density’s contrasts can be defined through the following equations, where subscripts “*E*” and “*P*” refer to the core and shell regions, and $\rho_{E}$, $\rho_{P}$, and $\rho_{s}$ are the core, the shell, and the buffer electron density, respectively:

(A1) $$\Delta \rho_{ES}=\rho_{E}-\rho_{s},\;\Delta \rho_{PS}=\rho_{P}-\rho_{s}.$$

The volumes of the whole micelle and of the core can be expressed as follows:

(A2) $$\;V_{M}=\frac{4\pi }{3}\frac{D_{M}}{2}{\left(\frac{D_{E}}{2\varepsilon }+R_{SH}\right)}^{2},\;\;V_{E}=\frac{4\pi }{3\varepsilon^{2}}{\left(\frac{D_{E}}{2}\right)}^{3},$$

where $D_{M}$ and $D_{E}$ = $\;D_{M}-2R_{SH}$ are the maximum sizes of the whole spheroid micelle and of its core along the polar axis, respectively. Here, ε = *R_pol_*/*R_eq_* is the ratio between the polar *R_pol_* and equatorial *R_eq_* radius of the micelle’s core, and $D_{M}$ = 2*R_pol_* + 2$R_{SH}$, $D_{E}$ = 2*R_pol_*, where *R_SH_* is the shell size. For prolate spheroids (ε > 1), $D_{M}$ coincides with the maximum size of the micelle, namely $D_{max}$. For oblate spheroids (ε < 1), $D_{M}$ = ε$D_{max}$.

For positive integers *n* and for a two-component spheroid-shaped micelle, the integrals given by Equation (1) have an analytical expression. We have already derived the solutions of Equation (1) for *n* = 1, 2, 4, 6 [14]. For *n* = 2, from Equation (1), we have the analytical expression for the gyration radius. For the *n =* 3 and *n* = 5 cases, we obtain the new formulae. Putting

(A3) $$a=\frac{D_{M}-2R_{SH}}{2},$$

(A4) $$b=\frac{D_{M}-2R_{SH}}{2\varepsilon },$$

(A5) $$a_{1}=\frac{D_{M}}{2},$$

(A6) $$b_{1}=\frac{D_{M}+2\left(\varepsilon -1\right)R_{Sh}}{2\varepsilon },$$

for *n* = 1, 2, 3, 4, 5, and 6 we obtain the following equations:

(A7) $$R^{1}=\frac{3\left\{ab^{2}\left(a+\frac{b^{2}ArcCos\left[a/b\right]}{\sqrt{b^{2}-a^{2}}}\right)\left(\Delta \rho_{ES}-\Delta \rho_{PS}\right)+a_{1}b_{1}^{2}\Delta \rho_{PS}\left(a_{1}+\frac{b_{1}^{2}ArcCos\left[a_{1}/b_{1}\right]}{\sqrt{b_{1}^{2}-a_{1}^{2}}}\right)\right\}}{8\left[a_{1}b_{1}^{2}\Delta \rho_{PS}+ab^{2}\left(\Delta \rho_{ES}-\Delta \rho_{PS}\right)\right]},$$

(A8) $$R^{2}=\frac{ab^{2}\left(a^{2}+2b^{2}\right)\left(\Delta \rho_{ES}-\Delta \rho_{PS}\right)+a_{1}b_{1}^{2}\Delta \rho_{PS}\left(a_{1}^{2}+2b_{1}^{2}\right)}{5\left[a_{1}b_{1}^{2}\Delta \rho_{PS}+ab^{2}\left(\Delta \rho_{ES}-\Delta \rho_{PS}\right)\right]},$$

(A9) $$R^{3}=\frac{ab^{2}\left(2a^{3}+3ab^{2}+\frac{3b^{4}ArcCos\left[a/b\right]}{\sqrt{b^{2}-a^{2}}}\right)\left(\Delta \rho_{ES}-\Delta \rho_{PS}\right)+a_{1}b_{1}^{2}\Delta \rho_{PS}\left(2a_{1}^{3}+10a_{1}^{3}b_{1}^{2}+15a_{1}b_{1}^{4}+\frac{15b_{1}^{6}ArcCos\left[a_{1}/b_{1}\right]}{\sqrt{b_{1}^{2}-a_{1}^{2}}}\right)}{16\left[a_{1}b_{1}^{2}\Delta \rho_{PS}+ab^{2}\left(\Delta \rho_{ES}-\Delta \rho_{PS}\right)\right]},$$

(A10) $$R^{4}=\frac{ab^{2}\left(3a^{4}+4a^{2}b^{2}+8b^{4}\right)\left(\Delta \rho_{ES}-\Delta \rho_{PS}\right)+a_{1}b_{1}^{2}\Delta \rho_{PS}\left(3a_{1}^{4}+4a_{1}^{2}b_{1}^{2}+8b_{1}^{4}\right)}{35\left[a_{1}b_{1}^{2}\Delta \rho_{PS}+ab^{2}\left(\Delta \rho_{ES}-\Delta \rho_{PS}\right)\right]},$$

(A11) $$R^{5}=\frac{ab^{2}\left(8a^{5}+10a^{3}b^{2}+15ab^{4}+\frac{15b^{6}ArcCos\left[a/b\right]}{\sqrt{b^{2}-a^{2}}}\right)\left(\Delta \rho_{ES}-\Delta \rho_{PS}\right)+a_{1}b_{1}^{2}\Delta \rho_{P}\left(8a_{1}^{5}+3a_{1}b_{1}^{2}+\frac{3b_{1}^{4}ArcCos\left[a_{1}/b_{1}\right]}{\sqrt{b_{1}^{2}-a_{1}^{2}}}\right)}{128\left[a_{1}b_{1}^{2}\Delta \rho_{PS}+ab^{2}\left(\Delta \rho_{ES}-\Delta \rho_{PS}\right)\right]},$$

(A12) $$R^{6}=\frac{ab^{2}\left(5a^{6}+6a^{4}b^{2}+8a^{2}b^{4}+16b^{6}\right)\left(\Delta \rho_{ES}-\Delta \rho_{PS}\right)+a_{1}b_{1}^{2}\Delta \rho_{PS}\left(5a_{1}^{6}+6a_{1}^{4}b_{1}^{2}+8a_{1}^{2}b_{1}^{4}+16b_{1}^{6}\right)}{105\left[a_{1}b_{1}^{2}\Delta \rho_{PS}+ab^{2}\left(\Delta \rho_{ES}-\Delta \rho_{PS}\right)\right]}.$$

Equations (A7), (A8), (A10), and (A12) have been already derived and discussed in [14]. This approach, indeed, can be considered as a fully analytical generalization of the graphical–analytical approach of [14]. The graphical estimates of *D*_max_ and *R*_SH_, derived from the derivative of the PDF, can be used as initial starting values for solving the following system of 6 equations (*n* = 1, …, 6) with the 5 unknowns ε, $\Delta \rho_{ES},$ $\Delta \rho_{PS},$ $D_{M}$, $R_{SH}$,

(A13) $$\eta^{\frac{n}{2}}R_{\mathrm{PDF}}^{n}=R^{n},\;\;\;n=1,\ldots ,6$$

where [14]

(A14) $$\eta =\frac{6R_{SH}D_{M}^{2}}{D_{M}^{3}-D_{E}^{3}}.$$

The overdetermined system of equations (more equations than unknowns) can be solved by searching the least squares associated with the solution of Equation (A13):

(A15) $$\mathrm{LSR}=\sqrt{\sum_{n=1}^{6}{\left(\sqrt[{n}]{\eta^{n/2}R_{\mathrm{PDF}}^{n}}-\sqrt[{n}]{R^{n}}\right)}^{2}}.$$

Odd exponents could lead to imaginary solutions in Equations (A7), (A9), and (A11). We need to select only the solutions for which the imaginary components are zero. Obviously, by calculating additional analytical expressions for high-order moments of the PDF, a number of equations larger than 6 could be used to constrain the above 5 unknown quantities. However, moments of much higher order could not add further independent constraints, with respect to moments of lower order, because they would be dominated only by contributions of higher distances.

The solution is a function of 5 independent quantities. This 5-dimensional space can be visualized by means of suitable bidimensional sections, choosing several pairs of these 5 variables. To do this, the SmoothDensityHistogram function of the software Mathematica 14.1 (Wolfram) can be used. It allows the user to plot a smooth kernel histogram of data. The option “Probability Density Function” can be also activated. In this way, we can calculate several bidimensional histograms of the 5-dimensional *LRS* function given by Equation (A15), calculated close to the minimum, as shown in the main text.

Moreover, we can calculate the PDF on a quantitative scale. Its integral for a two-component spheroidal micelle can be expressed as follows:

(A16) $$I\left(0\right)=4\pi \int_{0}^{D_{Max}}\mathrm{PDF}\left(r\right)dr=\frac{K}{N_{agg}}V_{M}^{2}{\left(\Delta \rho_{PS}+\left(\Delta \rho_{ES}-\Delta \rho_{PS}\right)\frac{V_{E}}{V_{M}}\right)}^{2},$$

where $V_{E}$, $V_{M}$ are the volumes of the core and the whole micelles, respectively. Here

(A17) $$K=\frac{\left(c_{mon}-cmc\right)N_{A}{r_{e}}^{2}}{MW_{mon}},$$

where the surfactant concentration $c_{mon}$ has been corrected by the critical micelle concentration *cmc*, both expressed in $g/{\mathrm{cm}}^{3}$, $N_{A}$ = $6.02214\cdot 10^{23}/\mathrm{mol}$ is the Avogadro number, $r_{e}$ is the classic radius of an electron $(2.81794\cdot 10^{-13}\;\mathrm{cm}$), $MW_{mon}$ is the molar mass of one monomer, and $N_{agg}$ is the average number of monomers aggregating into micelles [13]. For the micelle under study, K=  1.23 × 10^−7^ cm^−1^ electrons^−2^. Given the values of ε, $\Delta \rho_{ES},$ $\Delta \rho_{PS},$ $D_{M}$, $R_{SH}$ obtained by minimizing Equation (A15), we can use Equations (A16) and (A17) to calculate $N_{agg}$.

In the presence of the PSC, the question is how to estimate $N_{agg}$. For sample 3, in the presence of PSC in the buffer, $I\left(0\right)=0.085\;{\mathrm{cm}}^{-1}$. Thus, $\frac{I\left(0\right)}{K}≅831^{2}$. This is the average squared electron contrast per monomer of the micelles in solution, measured by SAXS. A PSC molecule has 232 electrons and a volume of 554 Å^3^. A water molecule has 20 electrons and a volume of 29.9 Å^3^, at room temperature [13]. Thus, a PSC molecule has a volume equivalent to 554/29.9 water molecules. Therefore, the squared electron contrast, due to a single PSC molecule, with respect to water, is ${\left(232-10\times 554/29.9\right)}^{2}≅47^{2}$. Since $47^{2}/831^{2}$~0.003, we can always use Equations (A16) and (A17) to calculate $N_{agg}$, even in the presence of PSC molecules in solution.

Generalizing the equation in ref. [20], the solvent fraction $x_{S}$ in the shell, ranging from 0 to 1, can be put as a function of $x_{PSC}$, and it can be estimated as follows:

(A18) $$x_{S}\left(x_{PSC},y\right)=\frac{\Delta \rho_{PS}+\rho_{H2O}-\rho_{sh}+x_{PSC}\times \left(1-y\right)\times \left(\Delta \rho_{PS}+\rho_{H2O}-\rho_{PSC}\right)}{\rho_{H2O}-\rho_{sh}+x_{PSC}\times \left(1-y\right)\times \left(\rho_{H2O}-\rho_{PSC}\right)}.$$

Here, $\rho_{sh}$ is the theoretical electron density due to the hydrophilic part of the monomer; $x_{PSC}$ is the ratio of the number of PSC molecules divided by the number of monomers inside the micelle ($x_{PSC}$ could be also greater than 1, because it is a ratio between the number of different molecules); *y* is the fraction of the PSC molecules per monomer inside the core and $\rho_{PSC}$ is its electron density; 1 − *y* is the fraction inside the shell. Even if $x_{PSC}$ = 0, for $\Delta \rho_{PS}=-\rho_{H2O}+\rho_{sh}$ in Equation (A18), we would have no solvent molecules inside the shell (no hydration of the shell). For $\Delta \rho_{PS}=0$, we would have only solvent molecules inside the shell (no shell). Therefore, actual $\Delta \rho_{PS}\;$values would fall between these two limits, due to the partial hydration of the hydrophilic part of the monomers and the eventual presence of PSC inside the shell.

An amount of 82.6 mg of TPGS in 19.9 g of solution (see Table 1) leads to 3.28 × 10^19^ monomers (55 μmol) in sample 1; 10 g of this solution was taken to realize sample 3. Thus, the number of monomers in 10 g of solution is *N_mon_* = 1.65 × 10^19^ monomers. Instead, the number of PSC molecules in the 10 g solution is *N_PSC_* = 1.09 × 10^19^ (18 μmol). Therefore, the ratio of *N_PSC_* and *N_mon_* in solution for samples 3 and 4 is *N_PSC_*/*N_mo_*_n_ ≅ 2/3 [13]. Thus, we should expect $x_{PSC\;}\leq 2/3$.

Moreover, between the hydrophobic and the hydrophilic parts of the monomers constituting the investigated micelles, we have a succinate linker, with z = 60 total electrons, which, added to the 536 electron of PEG 1000 and divided by the molecular volumes for succinate (123.6 Å^3^) plus PEG 1000 (1360.7 Å^3^), gives [13]

(A19) $$\rho_{sh}\;≅\;\frac{536+60}{123.6+1360.7}=0.402\;n_{e}/{Å}^{3}.$$

The above value is an estimate of the maximum electron density in the shell, for $x_{PSC}$ = 0, whereas for PSC we have $\rho_{PSC}=0.419\;n_{e}/{Å}^{3}$ [13].

Given $x_{S}\left(x_{PSC},y\right)$ by Equation (A18), the number of solvent (H_2_O) molecules inside the shell volume ($V_{M}$-$V_{E}$) can be estimated as follows:

(A20) $$N_{H2O}\left(x_{PSC},y\right)=x_{S}\left(x_{PSC},y\right)\times \frac{V_{M}-V_{E}-N_{agg}x_{PSC}V_{PSC}\left(1-y\right)}{V_{H20}}.$$

Given the total $\;N_{H2O}$ inside the shell, the number of PSC molecules $x_{PSC}$ per monomer into the micelle can be evaluated by the constraint that the sum of the volumes of all molecules inside the micelle—i.e., the solvent, monomer and PSC molecules—has to be equal to the micelle’s volume $V_{M}$:

(A21) $$N_{agg}x_{PSC}V_{PSC}+N_{agg}V_{mon}+N_{H2O}\left(x_{PSC},y\right)V_{H20}=V_{M}.$$

By inserting Equation (A20) into Equation (A21), we obtain the following equation:

(A22) $$N_{agg}x_{PSC}V_{PSC}+N_{agg}V_{mon}+x_{S}\left(x_{PSC},y\right)\times \left(V_{M}-V_{E}-N_{agg}x_{PSC}V_{PSC}\left(1-y\right)\right)-V_{M}=0,$$

with $x_{S}\left(x_{PSC},y\right)\;$given by Equation (A18). Equation (A22) can be solved numerically, after having determined $\varepsilon ,\;\Delta \rho_{ES},$ $\Delta \rho_{PS},$ $D_{M}$, $R_{SH}$, and $N_{agg}$, as previously described, to obtain a value for $x_{PSC}$ that depends on the fraction *y* of PSC molecules inside the core, as discussed in the main text.

## References

[1] Kabanov A.V., Alakhov V.Y. (2007). Micelles of amphiphilic block copolymers as vehicles for drug delivery. J. Control. Release.

[2] Torchili V.P. (2007). Micellar Nanocarriers: Pharmaceutical Perspectives. Pharm. Res..

[3] Lammers T. (2024). Nanomedicine and tumor targeting. Adv. Mater..

[4] Blanco E., Shen H., Ferrari M. (2015). Principles of nanoparticle design for overcoming biological barriers to drug delivery. Nat. Biotechnol..

[5] Discher D.E., Eisenberg A. (2002). Polymer vesicles. Science.

[6] Kinnear C., Moore T.L., Rodriguez-Lorenzo L., Rothen-Rutishauser B., Petri-Fink A. (2017). Form Follows Function: Nanoparticle Shape and Its Implications for Nanomedicine. Chem. Rev..

[7] Fang J., Nakamura H., Maeda H. (2011). The EPR effect: Unique features of tumor blood vessels for drug delivery, factors involved, and limitations and augmentation of the effect. Adv. Drug Deliv. Rev..

[8] Liu Y., Tan J., Thomas A., Ou-Yang D., Muzykantov V.R. (2012). The Shape of Things to Come: Importance of Design in Nanotechnology for Drug Delivery. Ther. Deliv..

[9] Meng F., Zhong Z., Feijen J. (2009). Stimuli-responsive polymersomes for programmed drug delivery. Biomacromolecules.

[10] Petros R.A., De Simone J.M. (2010). Strategies in the design of nanoparticles for therapeutic applications. Nat. Rev. Drug Discov..

[11] Verma A., Stellacci F. (2010). Effect of surface properties on nanoparticle-cell interactions. Small.

[12] Ghezzi M., Pescina S., Padula C., Santi P., Del Favero E., Cantù L., Nicoli S. (2021). Polymeric micelles in drug delivery: An insight of the techniques for their characterization and assessment in biorelevant conditions. J. Control. Release.

[13] De Caro L., Del Giudice A., Morin M., Reinle-Schmitt M., Grandeury A., Gozzo F., Giannini C. (2023). Small Angle X-Ray Scattering Data Analysis and Theoretical Modelling for the Size and Shape Characterization of Drug Delivery Systems Based on Vitamin E TPGS Micelles. J. Pharm. Sci..

[14] De Caro L., Stoll T., Grandeury A., Gozzo F., Giannini C. (2024). Characterization of Surfactant Spheroidal Micelle Structure for Pharmaceutical Applications: A Novel Analytical Framework. Pharmaceutics.

[15] U.S. Food and Drug Administration (2008). Drug Approval Package: Promacta (Eltrombopag) NDA#022291.

[16] Beth Wire M., Bruce J., Gauvin J., Pendry C.J., McGuire A., Qian Y., Brainsky A. (2012). A Randomized, Open-Label, 5-Period, Balanced Crossover Study to Evaluate the Relative Bioavailability of Eltrombopag Powder for Oral Suspension (PfOS) and Tablet Formulations and the Effect of a High-Calcium Meal on Eltrombopag Pharmacokinetics When Administered with or 2 Hours Before or After PfOS. Clin. Ther..

[17] Svergun D.I. (1992). Determination of the regularization parameter in indirect-transform methods using perceptual criteria. J. Appl. Crystallogr..

[18] Williams D.D., Peng B., Bailey C.K., Wire M.B., Deng Y., Park J.W., Collins D.A., Kapsi S.G., Jenkins J.M. (2009). Effects of food and antacids on the pharmacokinetics of eltrombopag in healthy adult subjects: Two single-dose, open-label, randomized-sequence, crossover studies. Clin. Ther..

[19] Rathod S., Bahadur P., Tiwari S. (2021). Nanocarriers based on vitamin E-TPGS: Design principle and molecular insights into improving the efficacy of anticancer drugs. Int. J. Pharm..

[20] Puig-Rigall J., Grillo I., Dreiss C.A., González-Gaitano G. (2017). Structural and Spectroscopic Characterization of TPGS Micelles: Disruptive Role of Cyclodextrins and Kinetic Pathways. Langmuir.

